# Reproducibility of native T1 mapping using ShMOLLI and MOLLI - implications for sample size calculation

**DOI:** 10.1186/1532-429X-18-S1-P2

**Published:** 2016-01-27

**Authors:** Anish N Bhuva, Sabrina Nordin, Heerajnarain Bulluck, Thomas A Treibel, Amna Abdel-Gadir, Stefania Rosmini, James C Moon, Charlotte Manisty

**Affiliations:** Barts Heart Centre, St Bartholomew's Hospital, London, UK

## Background

Native T1 mapping is becoming established to help diagnose and monitor myocardial disease.

The reproducibility of T1 mapping has not been well characterized, despite the important implications both for interpreting serial clinical studies, and for sample size calculation for surrogate endpoint in clinical trials. The SCMR consensus statement recommends measuring T1 in 2 imaging views. We investigated the test-retest reproducibility of two native T1 mapping techniques using different imaging views.

## Methods

51 subjects were scanned on two separate occasions within two weeks using a 1.5T (Siemens Avanto) scanner. This included 20 healthy volunteers (mean age 39 ± 8, 14 male) and 31 patients with disease that raises or lowers T1 (21 Fabry disease, 5 cardiac amyloidosis, 2 aortic stenosis, 3 cardiomyopathy, mean age 51 ± 15, 20 male) and 20 healthy volunteers. Native T1 maps were acquired in the four chamber (4Ch) and mid ventricular short axis (SAX) views using MOLLI 5s(3s)3s and ShMOLLI. Regions of interest (ROI) were drawn for both MOLLI and ShMOLLI. These were drawn on both views and by two independent observers on the two scans with blinding to minimise operator bias.

Inter-observer and inter-study (test-retest) reproducibility were assessed using ICC and correlation respectively, with sample size calculated using standard methods (Altman et al 1991).

## Results

Across all scans, there was a wide range of native T1 in this cohort. On average, ShMOLLI measured 54 ms lower than MOLLI (937 ± 81 ms vs 986 ± 68 ms, p < 0.001).

**Figure 1:**
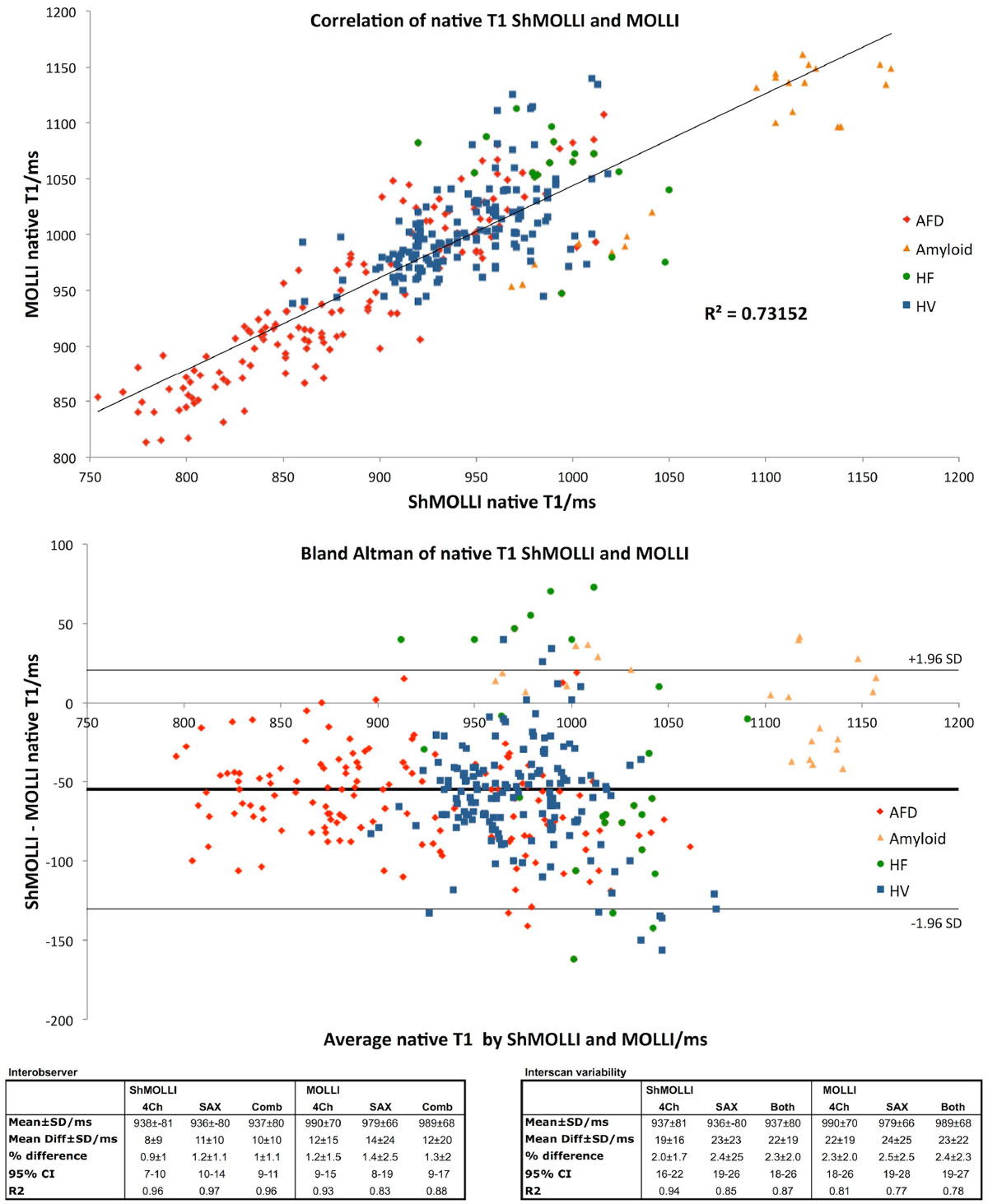
**Correlation and Bland Altman analysis of ShMOLLI and MOLLI across a range of patient groups**.

For all values, ShMOLLI and MOLLI correlated (R^2^ 0.73), but this correlation was not high (Figure 1).

Inter-observer reproducibility with both ShMOLLI and MOLLI was high, with no significant difference between techniques (ICC 0.98 and 0.94 respectively, p = NS).

Despite the limited correlation between ShMOLLI and MOLLI, test-retest reproducibility was very good with both techniques but better for ShMOLLI (R^2^=0.87 and R^2^=0.78 respectively, Z statistic -2.7, p < 0.008).

Native T1 values were not significantly different in the 4Ch or SAX views, for both MOLLI and ShMOLLI sequences, although there was a trend to lower test-retest variability in the 4Ch than SAX views for ShMOLLI (mean difference 19 ± 16 vs 23 ± 23 ms, p = 0.06).

**Figure 2:**
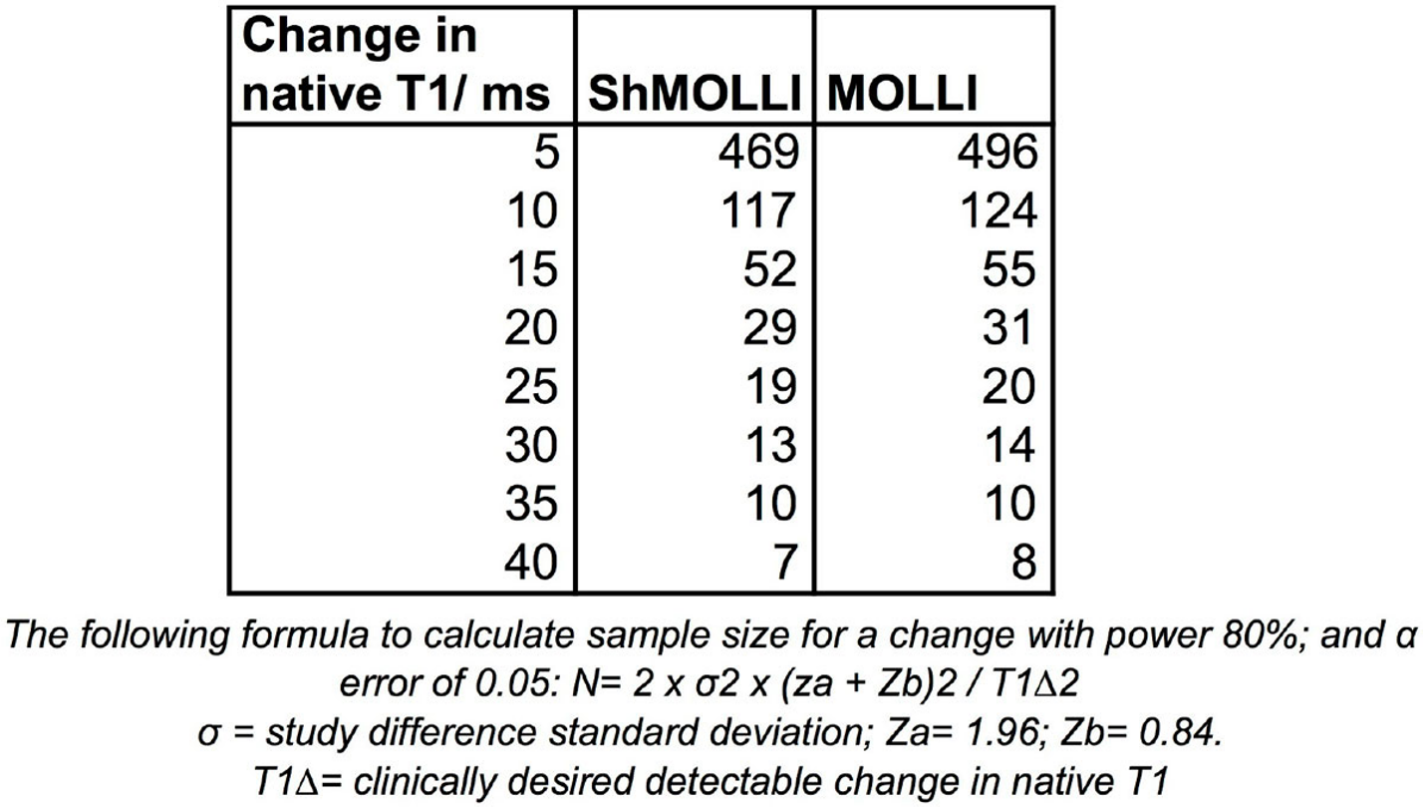
**Sample size calculations for ShMOLLI and MOLLI**.

Using these data, to detect a difference of 20 ms or 40 ms in native myocardial T1, a sample size of 29 vs 31 subjects (ShMOLLI versus MOLLI) or 7 vs 8 subjects would be needed. (Figure 2).

## Conclusions

In 51 patients/healthy controls with test: retest data at a single site, both MOLLI and ShMOLLI have excellent inter-observer and good test-retest reproducibility. The difference between ShMOLLI and MOLLI did not translate to an important difference in sample size estimates.

